# Knowledge and experience of cancer prevention and screening among Gypsies, Roma and Travellers: a participatory qualitative study

**DOI:** 10.1186/s12889-021-10390-y

**Published:** 2021-02-16

**Authors:** Louise Condon, Jolana Curejova, Donna Leeanne Morgan, Glenn Miles, Deborah Fenlon

**Affiliations:** 1 College of Human and Health Sciences, Swansea University, Singleton Park, Swansea, Wales, SA2 8PP UK; 2 Member of the Roma community, UK; 3 Member of the Gypsy community, UK

**Keywords:** Cancer prevention, Screening, Roma, gypsies and travellers, Qualitative, Peer researchers

## Abstract

**Background:**

The incidence of cancer is increasing worldwide, which has led to greater public health focus on primary prevention. Ethnic minorities have lower awareness of cancer risk factors and services, and are at greater risk of cancer mortality. While Gypsies, Roma and Travellers have poor health outcomes even in comparison with other ethnic minorities, little is known about how they view and enact primary prevention. This study takes a participatory approach to explore knowledge and experience of cancer prevention and screening in these communities.

**Methods:**

Peer researchers conducted interviews (*n* = 37) and a focus group (*n* = 4) with a purposive sample of community members in Wales and South-West England. Participants self-identified as Roma (from Slovakia and Romania) or as Gypsies, Travellers or Showpeople (here described as Gypsy/Travellers). A third of the sample were Roma, and a quarter male, with ages ranging from 18 to 77 years. Data were collected from October 2018 to March 2019.

**Results:**

Women and men knew that lifestyle factors, such as healthy diet, stopping smoking, drinking less alcohol and using sun protection, contribute to cancer risk reduction. However, there was a widespread lack of confidence in the effectiveness of these measures, particularly in relation to smoking. Traditional cultural beliefs were shared by Roma and Gypsy/Travellers, but did not necessarily affect the behaviour of individuals. Most women participated in cervical and breast screening but few Gypsy/Traveller men would engage with bowel screening, which conflicted with community ideals of stoical masculinity. Roma participants described language barriers to screening, with confusion about differences in timing and eligibility between the UK and Slovakian programmes; this led some to access screening abroad.

**Conclusion:**

This study provides new knowledge about how Gypsies, Roma and Travellers keep healthy and prevent disease, giving insights into similarities and differences between ages, sexes and communities. These culturally distinct and high-need ethnic minorities have specific needs in relation to cancer prevention and screening, which merit targeted and acceptable health promotion to reduce health inequalities.

**Supplementary Information:**

The online version contains supplementary material available at 10.1186/s12889-021-10390-y.

## Background

The incidence of cancer is increasing worldwide, which has led to greater public health focus on primary prevention. Effective interventions include reducing exposure to known causes of human cancer, such as smoking and excessive sun exposure; additionally national screening programmes facilitate early diagnosis and treatment [1]. Despite these measures, not all ethnic groups are equally protected against developing cancer, with some experiencing unequal access to information, screening and early diagnosis. In the United Kingdom (UK) it is recognised that lower awareness of cancer risk factors, symptoms and services exists among ethnic minority groups [2, 3], with socio-economically disadvantaged groups at greater risk of cancer incidence and mortality [4]. Prevention of cancer is the most effective way of protecting individuals and their communities, reducing physical and emotional suffering as well as health service costs.

Gypsies and Travellers are an ethnic group included in the census of England and Wales for the first time in 2011, when 58,000 people identified themselves as a Gypsy or Irish Traveller [5]. The inclusion of Roma people is recommended for the next census due to the disadvantage they experience and poor health outcomes [6]. In the UK the term ‘Roma’ denotes people from continental Europe with a Gypsy background, however, Roma is frequently used as a generic term for all Gypsies, Roma and Travellers in policy and research (e.g. European Agency for Fundamental Rights [7], Cook et al. [8]). Common characteristics of people with Roma heritage are a history of nomadism and a family-oriented culture, a distinct language and experiences of racism and discrimination. Around 6 million Roma people live in the European Union, making them the largest ethnic minority in Europe [7]. In the UK Showpeople, Boat People and New Travellers are often described as Travellers but are not included within the 2011 census definition of Gypsy or Irish Traveller.

In the UK data are not routinely collected within the National Health Service (NHS) for Gypsies and Travellers as an ethnic group, which is a barrier to knowledge about their health status [8]. Research has shown that Gypsies and Travellers have higher levels of ill health than the general population and lower life expectancy [9, 10]. They experience barriers in accessing health services [11], and underuse preventive services, including screening [12]. In their countries of origin Roma people commonly experience poor living conditions [13] and limited access to health services [14, 15]; in the UK they often live in overcrowded accommodation in the most disadvantaged areas [16]. Authorised and unauthorised Gypsy and Traveller sites in the UK are commonly situated in risky environments, such as beside busy roads or heavy industry [17]. A recent House of Commons report stated that Gypsies, Roma and Travellers have the worst outcomes of any UK ethnic group in education, health, employment, criminal justice and hate crime [6].

The incidence of cancer among Gypsies, Roma and Travellers is an ‘invisible’ health concern due to lack of data [8]. Parry et al. [9] found no inequality in self-reported rates of cancer; although van Cleemput [18] suggests this is due to lower life expectancy among Travellers. Cancer risk factors prevalent among Gypsies and Travellers include smoking, poor diet and increasing alcohol use among young men [19]. Among Roma, neoplasm is a frequent cause of death [20, 21]; risk factors include smoking, alcohol use, poor oral care [22] and obesity [23]. One Romanian study revealed low awareness of a national cervical cancer screening programme among Roma women, and uncertainty about their eligibility to participate [24]. The Leeds Gypsy and Traveller Exchange [25] describe barriers to bowel cancer screening and Jackson et al. [26] reported resistance among some Gypsies and Travellers to the human papilloma virus (HPV) vaccine for teenage girls, which protects against cervical and oral cancer.

Despite high levels of ill health and low life expectancy, there is little in-depth exploration of Gypsies, Roma and Travellers’ knowledge and experience of cancer prevention and screening. People from ethnic minority groups have unmet needs relating to the provision of cancer information, and there is a lack of knowledge about their experiences of cancer prevention [3]. Most current research focuses on cultural beliefs about cancer, some of which are shared with other ethnic minority populations [27]. Cancer is described as a taboo subject [28, 29], and some Gypsies and Travellers avoid saying the word, preferring euphemisms, such as ‘*that bad condition’* or ‘*that dirty thing’* [30]. Fatalistic beliefs, allied to stoicism and lack of trust in health professionals, are common among Gypsies and Travellers [12, 28, 29, 31]. Cancer fatalism is more common in people of lower socio-economic and educational status, and acts as a deterrent to receiving optimal services and treatment [32, 33]. The aim of this study was to explore knowledge and experience of cancer prevention and screening among Gypsies, Roma and Travellers.

## Methods

### Design

A qualitative and participatory approach was taken to explore in depth this culturally sensitive topic. Roma and Gypsy/Traveller people often lack trust in outsiders and would be unlikely to discuss sensitive subjects with people outside their own community [31]; to address this all interviews were conducted by peers who had received training in qualitative data collection from the PI. Due to the taboo nature of the subject peer researchers recruited only people who they knew would be willing to discuss cancer, which meant that interviewees were drawn primarily from their extended family and acquaintances. A purposive sampling method was used to select Roma and Gypsy/Travellers, of a range of ages and both sexes. This diversity was facilitated by peer researchers themselves being diverse in gender, age, nationality and ethnic subgroup, which contributed to a range of opinions being accessed. Semi-structured interviews are a flexible method of generating naturalistic data [34]. In a second phase of the study workshops were conducted with community members and professionals (third sector and from local public health organisations) to co-produce interventions to increase cancer awareness and prevention.

### Recruitment

Peer researchers identified, recruited and obtained consent from participants. The six peer researchers were aged 18–58 years; one was Roma and the remainder Gypsy/Travellers, four female and two male. Three peer researchers were recruited by an outreach worker from Travelling Ahead (a Welsh charity providing advice and advocacy for Gypsy, Roma and Traveller communities), and three were known to the PI from previous research. As all peer researchers had additional paid and unpaid work commitments, researchers varied in the numbers of participants they could recruit and interview (range 1–11, mean 6 interviews). Targets were set for a third of participants to be Roma, and a third male as both groups are often underrepresented in qualitative research. Almost all participants were known to peer researchers prior to interviews, and very few declined to participate; the most common reason for non-participation was lack of time. Peer researchers provided information (face-to-face) about the project one week in advance of the interview, using translated information sheets. Recruitment sites were in South and West Wales, and South West England. Participants were offered a supermarket voucher as a ‘thank you’ for their time.

### Data collection

Interviews were conducted by peer researchers, face-to-face in participants’ choice of setting, (most often at home); they lasted between four and 37 min (mean 15 min) and were audiotaped. Romanian participants, who were all street newspaper sellers, requested to be interviewed jointly. This focus group (40 min) was conducted in a private room in the office of the street newspaper and audiotaped. No field notes were kept, but after interviews peer researchers participated in audiotaped debriefs about the experience. The post-interview debriefs with the academic team and peers promoted reflection and an opportunity to ensure data collection was consistent across researchers.

Although the target for male participants was not reached, data collection finished at 41 interviews as it was not possible to extend the project timescale. Questions were devised in consultation with peer researchers and focused on healthy lifestyle, uptake of routine screening and access to information about cancer within communities. Peer researchers practised using the topic guide as part of their training, no changes were subsequently made. Additional File 1 contains the full topic guide which was developed for this study and used for interviews and the focus group.

Roma participants were interviewed in either Romanes (Romany language) or Slovak according to participant preference. The Roma interviewer led the focus group for which a professional Romanian interpreter translated contemporaneously. Qualitative interview data were transcribed verbatim. Interviews conducted in Romanes and Slovak were transcribed and then translated by a professional translator. A sample of transcripts were checked against the audio-recording by a second translator, as has been done in previous studies [26, 35] to enhance rigour. Transcripts were not returned to participants for comments, as not all could read English.

### Data analysis

Data analysis was guided by the Framework Approach [36], which addresses applied social questions and has been used previously to identify health issues in Gypsy-Traveller populations [26, 37]. This approach provides a systematic and flexible way of managing large amounts of qualitative data from which themes can be derived both deductively and inductively [38]. Following familiarisation with the data, a sample of interview transcripts were used to develop a thematic framework, then systematically applied to the whole data set in Excel by three professional data coders. LC and DF grouped all themes into higher order labels, and peer researchers reviewed the thematic analysis to ensure that the interpretation reflected the meaning and context of interviews. In analysis, Roma participants from Slovakia and Romania were grouped together as Roma, and those who self-identified as Gypsies, Travellers, Gypsy-Travellers or Showpeople were grouped as Gypsy/Travellers.

## Results

Table 1 provides demographic details of the 41 participants. Education levels were higher for Slovakian Roma participants, but over 40% of the total sample had no qualifications. As in the 2011 census [5], 60% of Gypsies and Travellers in this study had no qualifications; this compares with 23% for the whole population in England and Wales without qualifications. All Roma were housed, while the majority of Gypsies and Travellers lived in caravans or chalets. It is known that Roma people predominantly live in rented accommodation in the UK [6], but more Gypsy/Travellers in this sample (68%) lived on caravan sites than in the 2011 census cohort (24%) [5]. Ethnicity was self-defined; most non-Roma described themselves as Gypsies, some as Travellers and a minority as Gypsy/Travellers or Showpeople (who travel with fairs and circuses). The final sample included 12 young people (18–25 years), 16 adults (26–50 years), and 13 people aged over 50 years. A quarter of the sample (11/41) lived in England, the remainder in Wales. All Roma direct quotes are translated from Romanes or Slovak.

**Table 1:** Demographic details of participants (*n* = 41)

Self-defined ethnicity and nationality	Sex	Age	Type of housing	Highest educational qualification
**Gypsies and Travellers (Welsh)** (*n* = 20)	Female: 14 Male: 6	Range 18–71 years (median 35)	Caravan/chalet: 14 Housed: 6	No qualification: 11 GCSE: 3 Diploma: 3 Bachelor degree: 2 Not stated: 1
**Gypsies and Travellers (English)** (*n* = 5)	Female: 4 Male: 1	Range 30–77 years (median 69)	Caravan/chalet: 3 Housed: 2	No qualification: 4 Diploma: 1
**Showpeople (English)** (*n* = 2)	Female: 2	Range 22–49 years	Caravan/chalet: 2	No qualification: 1 GCSE: 1
**Roma (Slovakian)** (*n* = 10)	Female: 7 Male: 3	Range 19–58 years (median 29)	Housed: 10	GCSE: 7 Master’s degree: 1 A-level: 1 Not stated: 1
**Roma (Romanian)** (*n* = 4)	Female: 3 Male: 1	Range 19–28 years (median 22)	Housed: 4	No qualification: 2 GCSE: 2
**Total (*n*** **= 41)**	**Female: 30** **Male: 11**	**Range 18–77 years**	**Housed: 22** **Caravan/chalet: 19**	**No qualification: 18** **GCSE: 13** **Diploma: 4** **Bachelor degree: 2** **Not stated: 2** **A-level: 1** **Master’s degree: 1**

Key

*GCSE* General certificate of secondary education (school-leaving exam at age 16 years)

*A-level* Advanced level (exam at age 18 years)

*Diploma* Higher Education award below the standard of a Bachelor’s Degree

In reporting findings the participant’s number is given (i.e. P1- P41), the self-defined ethnicity, sex ([M] ale or [F]emale) and age in years.

Thematic analysis of interview data led to the identification of three main topic areas: ‘Beliefs about cancer’, ‘Reducing the risk of cancer’ and ‘Cancer screening’. Three main themes were identified deductively (from the specific research question and previous literature as well as these data) whereas cross-cutting themes (which reflect participants’ experience and their ascribed meanings) emerged inductively from open coding and subsequent refinement by team members. A summary of the interrelationship between main and cross-cutting themes is given in Table 2.

**Table 2:** Summary of relationship between main and cross-cutting themes^a^

Cross-cutting themes	Main themes		
	Beliefs about cancer	Risk reduction in daily life	Participation in screening
**Ethnic identity**	Aware of traditional views Mistrust of health promotion messages	Aware of ‘healthy lifestyle’ messages Activities with children and animals	Belief that community uptake is low Screening outside NHS (e.g. abroad (*Roma only*) or via private medical services in UK (*Showpeople only*))
**Gender roles**	Men are stoical about illness Men and women do not discuss private health matters	Women provide meals Women and men’s exercise is highly gendered	Women likely to attend screening Men unlikely to attend
**Fatalism**	Traditional cancer fatalism	Doubt whether prevention is possible	Men likely to await symptoms
**Fear of being shamed**	Tradition that cancer is taboo	Low health literacy	Exposure of body contravenes privacy (*Gypsy/Travellers only*)
**Social and economic factors**	Low literacy levels Language barriers (*Roma only*)	Expectation of early morbidity Awareness of risk in work and living conditions	Lack of familiarity with UK screening system (*Roma only*)

^a^Themes apply to all ethnic minority subgroups unless specified

### Beliefs about cancer

A strong sense of ethnic identity coloured participants’ beliefs about cancer, whether they themselves held these beliefs or not. Both Roma and Gypsy/Travellers could describe ‘what people from my community think about cancer’. Participants described cancer as a taboo disease that was not spoken of outside the family, and even concealed from others within the community. Phrases used included ‘*the big C’*, ‘*the bad disease*’, ‘*that undercover thing’* and ‘*the Devil’s disease’*, and participants of all ages, including teenagers, adopted these euphemisms. Both Roma people and Gypsy/Travellers referred to cancer as an evil disease, latent in everyone, which could be disturbed and become active. Table 3 shows dominant cultural beliefs about cancer which were shared by people from both Roma and Gypsy/Traveller backgrounds. While both Roma and Gypsy/Travellers spoke of cancer as taboo, only Roma participants described avoiding people with cancer.

**Table 3:** Beliefs about cancer shared by Roma and Gypsy/Travellers

	**•Cancer is an evil disease**
**Roma**	*‘It is a malicious sickness.’* P4-Roma, F53
**Gypsy/Travellers**	*‘It’s our big, black devil that nobody wants to mention.*’ P12-Traveller, F60
	**•Cancer is taboo**
**Roma**	*‘One does not even want to hear about that sickness, or … even want to be in contact with those people.’* P24-Roma, F29
**Gypsy/Travellers**	*‘It’s sort of viewed as taboo … people just don’t want to call it the cancer word.’* P8-Gypsy, M18
	**•Fear of cancer**
**Roma**	*‘Everyone is the most afraid of cancer.’* P1-Roma, F28
**Gypsy/Travellers**	*‘Gypsies seem to think if you’ve got that, you are going to die.’* P7-Gypsy, F55
	**•Cancer is latent in everyone**
**Roma**	*‘I think that all of us have it. And the one who does not protect himself and does not try to be healthy, in that case it will simply be confirmed.’* P26-Roma, M29
**Gypsy/Travellers**	*‘Every person has a cancer inside of them, it just needs to be awakened.’* P41-Showperson, F49
	**•Screening is avoided**
**Roma**	*‘They should go regularly, but the people neglect it.’* P27-Roma, M58
**Gypsy/Travellers**	*‘They’re stubborn and they’re ignorant to it because the reason is they’re scared, they don’t want to know things...when... older people was … younger there was no such thing.’* P21-Gypsy, F49

Although they could describe traditional beliefs both men and women said individual attitudes towards cancer were changing. Not all considered cancer a death sentence: ‘*I mean if cancer’s in your body you’re not gonna die from it just like the older generation think.*’(P36-Gypsy/Traveller, F38). Both Gypsy/Traveller and Roma participants spoke of the possibility of cure if cancer was diagnosed early and treated effectively. Cancer did not hold the pre-eminent position as the most fatal and worrying of diseases. Coronary heart disease, diabetes and mental ill health were described as being highly prevalent in the Gypsy/Traveller community, but were not described as taboo or with connotations of evil. A common view was that disease is an inevitable part of ageing: ‘*A lot of people get to about 40s and they do have diseases … heart disease, cancer’* (P23-Gypsy, F18).

While recognising that superstitions about cancer continued to exist in the community, one older man stated that cancer was simply a disease like any other and there was no reason to avoid saying the word (‘*If you break your arm you say you broke your arm. So if you’ve got cancer, you’ve got cancer, in my opinion.’* P19-Gypsy, M73). Beliefs such as thinking that saying the word risked causing the disease, were described as old-fashioned and untrue by both Roma and Gypsy/Traveller participants:

*‘Cos I think, lately, all the Travellers have come on, haven’t they? Like way before, they wouldn’t … talk about nothing … Oh, you mustn’t say that, cos it’ll come on you. All things like that. They don’t take no heed no more.’* P20-Gypsy, F64.

A few Gypsy/Travellers displayed highly inconsistent attitudes to cancer, sometimes making contradictory statements (e.g. cancer is incurable/cancer can be treated if caught early) during the course of one interview; thus individual attitudes could be changeable as well as fixed.

Language and literacy were obstacles to accessing health information about cancer from outside the community, and many relied on family and friends for knowledge. Cancer patients were viewed as good sources of information by Roma and Gypsy/Traveller people, although several younger Gypsy/Travellers suggested this knowledge was inaccurate because the older generation did not have a tradition of using health services. Health professionals were considered the best source of knowledge, and some participants were confident they would be listened to and receive helpful information. Barriers to understanding information from health professionals were medical terminology which was hard to understand and accompanying feelings of shame; ‘*It’s embarrassment of have to feeling dumb, feeling stupid, they knows big posh words where we don’t know big posh words.’* P11-Gypsy/Traveller, F48). Some accessed information via the internet, although this information was recognised as potentially misleading. NHS websites were highly trusted.

### Reducing the risks of cancer

Lifestyle measures to keep well and avoid disease were described as eating healthily (more fruit and vegetables, less meat and fat), taking exercise (gym, family activities with children and care of animals) and use of sun protection. Traditional Gypsy/Traveller pursuits such as horse-riding and boxing were enjoyed. Behaviour change was mentioned in relation to lifestyle; for instance, sun protection had not been used in the past, but was now seen as important by both men and women, particularly for children. Sun beds were viewed as risky, and one woman had given up using them after seeing warnings in salons. Three participants mentioned HPV vaccine for teenage girls as protection against cervical cancer.

Exercise was often related to gendered work, frequently childcare /housework for women and manual labour for men. A small number of people were unable to exercise due to disability, such as respiratory disease and arthritis. For Showpeople (men and women) their work was a form of exercise: ‘*We operate the rides and pull them down and build them up, that keeps us fit and active*’(P31-Showperson, F22). Outdoor work was recognised as a skin cancer risk and one man had been exposed to carcinogenic materials in the building trade:

*‘Working in asbestos stuff, and stuff like that, that is severely cancerous stuff … there’s power stations and lagging and all that … that’s all asbestos. So, it’s floating round dust. You’re breathing that into your lungs all the time.’* P14-Traveller, M71.

Nutrition was the most commonly suggested way of keeping healthy and preventing disease. Both Roma and Gypsy/Traveller participants referred to eating ‘five a day’ to increase fruit and vegetable consumption, and one woman (aged 77 years) mentioned that breastfeeding protects mothers against cancer. Women were the primary providers of meals, and attempted to make these healthy, for instance by concealing extra vegetables in children’s meals. Several participants described making changes to their diet in response to advice from health professionals. A Roma woman had limited family portion sizes and fat following advice from a cardiologist, and diabetic care nurses were also a source of dietary advice.

Despite clear ideas about protective and risk factors, many fundamentally doubted that healthy lifestyle choices would protect an individual from cancer, preferring to attribute disease to fate, genetics or God’s will. This was most marked in relation to smoking. Many participants (Roma and Gypsy/Travellers) described themselves as current smokers; some wished to quit but stated they could not due to addiction. Others continued as long as they were not experiencing any worrying symptoms: ‘*When you simply do not have an experience with it, then your brain does not tell you to quit for the reason not to get cancer*’ P3-Roma, M23. Table 4 shows the common arguments given to suggest that cancer is caused by factors other than smoking. Such doubts led to disbelief in health promotion messages:

*‘People say … leaflets have said that you should stay out of the sun, don’t smoke, reduce alcohol, and exercise. But, personally, I don’t think they’re right because I know many people that have a great weight and exercise a lot, and they still could die from having cancer.’* P22-Gypsy, F22

**Table 4:** Arguments given to explain why smoking does not cause cancer

**Cancer is God’s will**	*‘Some say that the people who smoke, get it, right? I myself think that it comes from God. If he wants us to be sick, we will be.’* P1-Roma, F28
**Children can develop cancer**	*‘I know a young girl that, um, had quite a healthy lifestyle, very young girl and got Stage 4 … it happens in children as well, so, they’ve never smoked, they’ve never drunk, they’re not overweight … So, we just don’t know do we?’* P17-Gypsy, F30
**Cancer is due to genetics**	*‘I think it’s hereditary. They says it ain’t but it is.*’ P15-Gypsy, F69
**Non-smokers can develop cancer**	*‘At least four to my knowing, maybe more, of my own family and my husband’s family were non-smokers, and only had a drink over Christmas time, and they all died with cancer.’* P12-Traveller, F60
**Stopping smoke can cause cancer**	*‘My auntie stopped smoking, she died a couple of months after with lung cancer … So, her smoking never caused it.*’ P13-Gypsy/Traveller, M21
**Evidence is inadequate**	*‘There is a few things that actually can cause but it’s not proven, such as smoking.*’ P23-Gypsy, F18

Several participants exhibited a lack of confidence in what they were told by authorities, preferring to make their own judgements according to their own experience. The risks of Gypsy/Roma/Traveller daily life (relating to poverty, work and housing) played a part in eroding messages about keeping healthy. When risk was a major part of life, unhealthy behaviours such as smoking were of lesser importance because ‘*your risk factors is, is going to kill you at one point*’ (P13-Gypsy/Traveller, M21). This was most apparent in the young Romanian Roma group, who saw health as being able to eat and work, and that illness was as result of factors such as street sleeping.

### Uptake of screening

Participants frequently stated that members of their community (Gypsy/Traveller or Roma) would be unlikely to attend screening, but that they themselves did attend. Older participants described a generational change with younger people being more likely to attend. Almost all eligible women stated they took up cervical screening, and some had had mammograms. One participant described women in the Showpeople community paying privately for annual screening, because they needed to remain healthy to run their businesses. Accounts of screening were generally positive and staff were described as kind. A minority avoided screening because they feared diagnosis, but experience of cancer within the family was cited as a motivating factor.

Among Roma people who had experience of screening abroad, there was confusion about the timing of screening in the UK. More frequent screening was available in Slovakia to those who had health insurance, and women in their early 20s had had cervical screening (available from 25 years in the UK) and mammograms (available from 50 years in the UK). Because screening in Slovakia was seen as more comprehensive, some chose to access screening abroad. Not speaking English was a barrier to attending screening in the UK. Non-English speakers invariably described using family members to translate rather than requesting or being offered interpreters:

*‘I used to go when I was at home … but after I came here...I tell you the truth, I do not even feel like going, you know? Because my English is not good, and dragging always someone along with me to translate for me … I have a sister-in-law who speaks English well, or the cousin of my husband, he speaks well, too. So usually them … .but he lives in* [city] *so I do not really want always to ask him to travel up to here, and my sister-in-law has a small daughter*.’ P1-Roma, F28

Both Roma and Gypsy/Traveller women thought cervical screening was offered too late in the UK. For Gypsy/Traveller women this was because they were likely to be married with children by the age of 25 years, and also because they knew of celebrities who had died young of cervical cancer. As a private matter, screening would not be discussed between the sexes.

Screening could conflict with the ideals of privacy and modesty that are part of the Gypsy/Traveller culture. Although most overcame these feelings, for some it continued to be a barrier even when screening was offered opportunistically in primary care:

*‘They do say to you … is your smear up to date? I’m like no, and then they’ll say well, um, have you got time to do it now? I’ll always make an excuse and say no, because it’s really intrusive and I do feel uncomfortable doing it.’* P36-Gypsy/Traveller, F38.

For some, increased age and experience of the healthcare system did not serve to alleviate cultural distaste for intimate procedures. This was considered poorly understood by health professionals; ‘*Settled people don’t think it’s shameful, but you do and you’ve had so many children they think you can get over the shame-ness, but it don’t*’ (P11-Gypsy/Traveller, F48).

Gypsy/Traveller women described men as not attending medical appointments because of cultural stereotypes of masculinity and stoicism. To some extent this was borne out by male participants. The only universal screening for men in the UK is bowel screening, which was perceived by both sexes as the most repugnant procedure. One man, who previously described himself as generally taking medical advice, was aware of bowel screening but said he would attend only if he thought something was wrong, adding, ‘*Most men just don’t want to entertain it’* (P14-Traveller, M71). In both communities a minority of participants said that they would not go for screening unless they had symptoms, denoting a misunderstanding of the concept of screening.

Prostate cancer was not included in the topic guide as there is no UK screening programme, but one man raised this. He highlighted the particular challenges for a Gypsy/Traveller man attending.

*‘On the site I grew up, if a man got himself checked there would be jeering afterwards … I think men just might go … and say I've got a bit of a bug or a cold or something or, and then see the hospital or doctors.*’ P39-Traveller, M33

This demonstrates the very strong cultural views about masculinity and privacy, and a real fear of the opinion of other men if these boundaries were transgressed.

## Discussion

This was the first study to explore both Roma and Gypsy/Travellers’ views on primary cancer prevention. Cultural beliefs about cancer (such as cancer is contagious [39] and avoiding the word [40]) were reported in this study by Gypsy/Travellers and Roma. Cancer was described as an unmentionable and feared disease [28, 29, 31] by both groups, and as an evil disease which is latent in everyone. While individual participants might not personally hold a belief, they considered such ideas were prevalent and continued to influence health behaviours, such as attending screening. This supports the view that a strong ethnic identity and coherent cultural beliefs underpin Travellers’ health-related behaviour [12]. However, this study demonstrates that while traditional beliefs form the backdrop to views about cancer (for individuals of all ages), superstition about cancer is declining. Jackson et al. [26] reported changing attitudes among younger Travellers who have greater contact with health professionals, findings which were partially mirrored here. Here, age was not the major determinant of attitudes but contact with trusted health professionals was effective in modifying views. Fallacious beliefs, such as cancer being contagious, were mainly expressed in this study by the poorest (street newspaper sellers) who had little exposure to health services.

This study revealed greater knowledge of lifestyle-related causes of cancer among Gypsy/Travellers than previous research. Examples of healthy lifestyle behaviours largely accorded with public health messages to reduce disease [41, 42]. Women’s activity, based on housework and childcare, was unlikely to be of sufficient intensity to lower cancer risk [43]. Most factors known to increase the risk of cancer were cited by women and men, and some subsequent behaviour change described, most commonly in relation to healthy eating. Berlin et al. [29] reported ignorance about the risks of sun bed use and alcohol in a predominantly female sample of Gypsies/Irish Travellers in England; however, in this study almost all women, and some men, described using sun protection (particularly for children), and alcohol was cited as a risk factor. Men’s knowledge of the risks of sun exposure during outdoor work appeared better than some comparable groups [44]. The greater knowledge of cancer risks demonstrated in this study suggests increased awareness of, and receptivity to, health promotion messages among Gypsies, Roma and Travellers.

Smokers and non-smokers were aware that smoking is linked to the development of cancer, which accords with the findings of Bekalu et al. [45] that awareness of the harms of smoking does not necessarily lead to behaviour change. Young and old participants sought to minimise the adverse consequences of smoking, and even those who led the ‘healthiest lifestyles’ tended to fall back on old beliefs about fate and chance. Kelly et al. [46] point out that smoking is part of people’s everyday life, ingrained in social interaction and linked to personal identity; hence it is difficult (and for some impossible) to change. Smoking is recognised as a coping activity used by people with little control over other risk factors in their lives [47], and this is likely to apply to Roma and Gypsy/Traveller communities who commonly experience poverty and marginalisation [18]. Notably no participant (Gypsy/Traveller or Roma) mentioned the consequences of passive smoking, although passive smoking in childhood increases the risk of cancer in adulthood [48]. Lung cancer is one of the most common but preventable cancers [49], so targeted health promotion is merited in these communities.

Barriers to screening participation differed between Roma and Gypsy/Travellers. Slovakian Roma people were familiar with screening programmes prior to migration, and considered the UK offer less comprehensive. As in previous studies of European migrants [50–52], language difficulties and poor knowledge of the UK health system were barriers to service use. Current guidance on interpreting and translation in the NHS states that non-English speakers should not be required to provide their own interpreter [53], but in this study lack of interpreters was a barrier to accessing services. For Gypsy/Travellers screening contravened values of modesty and privacy, but as in the general population [54], women had greater acceptance of screening. For men, ideals of masculinity, specifically stoicism, influenced and constrained their individual healthcare choices. Health promotion for men is therefore recommended, but is likely to be complicated by the high value community members place on their Gypsy/Traveller identity and the shared beliefs, values and ideals that accompany this.

Smith and Ruston [55] suggest that many Gypsies and Travellers are over-reliant on community members as a source of health advice, which exacerbates the risks of ill health. This study suggests that health knowledge is readily available from family and friends within Gypsy/Traveller communities, but is often considered unreliable because past generations did not engage with health services. Doctors were most highly valued as sources of advice, although as in previous studies [35, 56] their language could be perceived as alienating. Common barriers to accessing information are low literacy and lack of trust [57] and in reaction to this, Gypsy/Travellers can be quick to believe that they are offered misinformation [18]. For most cancers avoidance of risk would have to occur decades before the onset of symptoms [58], which means that evidence from personal observation and experience is likely to mislead. In communities with high morbidity and mortality, motivation to take action to prevent cancer throughout the life course is likely to be lower than in affluent communities with high expectations of long life. This study confirms that socio-economic factors play a large part in shaping attitudes to risk reduction among Roma and Gypsy/Travellers, and in creating barriers to using preventive health services.

### Strengths and limitations

The purposive sample included a variety of age groups, Roma and men, which increased validity, and resulted in more nuanced findings than previous studies of Gypsy/Travellers’ attitudes to cancer. The inclusion of men presented their view first-hand, in addition to women’s reports of men’s attitudes and behaviour. Data collection by peer researchers ensured that participants were prepared to discuss this sensitive and culturally taboo subject. However, participation was primarily limited to extended family and acquaintances of peer researchers, who may be less representative of these ethnic minorities as a whole. More participants lived on caravan sites than among Gypsy/Travellers in general, but none were ‘roadside’ (habitually nomadic) travellers, which limits generalisability to this group. The challenges of recruiting participants from groups defined as ‘hard to reach’ are well recognised [59, 60]. As Cook et al. [10] have pointed out, Gypsies, Roma and Travellers are diverse, differing in language, customs, housing and ethnic self-identification. What is remarkable in the light of these differences is that Roma and Gypsies/Travellers in this study shared traditional beliefs about cancer, were similarly ambiguous about the possibility of prevention, and experienced barriers to screening.

## Conclusion

This study has provided new knowledge about how Gypsies, Roma and Travellers keep healthy and prevent disease, giving insights into similarities and differences in their attitudes and beliefs. Health needs differed between communities (Gypsy/Traveller and Roma, Slovakian Roma and Romanian Roma), and these differences were most marked in relation to screening. Use of interpreters is vital to ensure non-English speaking Roma are able to engage with health services. The study shows how traditional attitudes serve to compound socio-economic factors to justify engaging in risky health behaviours and to reduce the likelihood of screening participation, particularly among Gypsy/Traveller men. To facilitate effective health promotion, relationships of trust need to be established between service users and providers. Without improvement in the societal circumstances of Gypsies, Roma and Travellers, morbidity and mortality are unlikely to decrease and community expectations of health will remain low. Improving the conditions into which people are born, live and work is the foundation for addressing the wider determinants of health.

## Supplementary Information

**Additional file 1:.** Topic guide for interviews and focus group.

## Data Availability

The datasets generated during the current study are not publicly available due to the conditions of ethical approval.

## Abbreviations

F: Female
HPV: Human papilloma virus
M: Male
NHS: National Health Service
P: Participant
PI: Principal investigator
UK: United Kingdom
