# Supplementary material for: Knowledge and experience of cancer prevention and screening among Gypsies, Roma and Travellers: a participatory qualitative study
Source: BMC Public Health. 2021 Feb 16;21:360. doi: 10.1186/s12889-021-10390-y (PMC7885498; doi:10.1186/s12889-021-10390-y)
Supplement: Supplementary file 1 — Additional file 1:. Topic guide for interviews and focus group. [file 12889_2021_10390_MOESM1_ESM.pdf]

Topic guide (with prompts)

1. Tell me about what you do to keep yourself (and your family) well?
    a. Diet
    b. Exercise
    c. Anything else? (please describe)

2. What sort of bad diseases do Gypsies/ Travellers/ Roma/Showpeople worry about?
    a. Heart disease
    b. Cancer- what sort?
    c. Anything else? (please describe)

3. How is cancer viewed by you, and those in your community? Are there any other words used?

4. Do you know of anyone who has had cancer and could you tell us about it?
    a. Diagnosis (NB screening)
    b. Care- health professionals/family

5. What do Gypsies/ Travellers/ Roma/Showpeople think about the screening? Do they go for screening (alone or who with)? Have you any stories about screening?
    a. GP over 50 health check
    b. Breast screening
    c. Cervical screening
    d. Bowel screening (use home kit)
    e. Prostate check at GP surgery

    6. Is there anything people can do to reduce the risk of cancer?
        a. Use sun protection, stop smoking, Reduce alcohol, Healthy diet, Healthy weight, Exercise, HPV vaccination
        b. Seek medical help for symptoms (e.g. persistent cough, headaches, weight loss and unusual/persistent pains)
        c. Anything else? (please describe)

    7. Has anyone ever given you information about how to keeping healthy and reducing the risks of cancer? If so who?
        a) Friend
        b) Family (who?)
        c) Health professional (who?)
        d) Have received written information (who from and what about?)

8. Who would you go to if you wanted more knowledge about cancer and how to prevent it?
    a. Family
    b. Friends
    c. Health professionals
    d. TV programmes (which?)
    e. Online (which websites?)
    f. Anything else? (please describe)

9. Thank you and is there anything you would like to tell us/ask?
